# Identification of a Triple-Reassortant H1N1 Swine Influenza Virus in a Southern China Pig

**DOI:** 10.1128/genomeA.00229-14

**Published:** 2014-03-27

**Authors:** Zhixun Xie, Minxiu Zhang, Liji Xie, Sisi Luo, Jiabo Liu, Xianwen Deng, Zhiqin Xie, Yaoshan Pang, Qing Fan

**Affiliations:** Guangxi Key Laboratory of Animal Vaccines and Diagnostics, Guangxi Veterinary Research Institute, Nanning, Guangxi Province, China

## Abstract

We report here the complete genome sequence of a triple-reassortant H1N1 swine influenza virus strain, A/swine/Guangxi/BB1/2013 (H1N1) (GXBB1), isolated from a swine in the Guangxi Province of southern China in 2013. We obtained the complete genome sequence of the GXBB1 virus. Sequence analysis demonstrated that this H1N1 virus was a triple-reassortant swine influenza virus (SIV) whose genes originated from avian, human, and swine, respectively. Knowledge regarding the complete genome sequence of the GXBB1 virus will be useful for epidemiological surveillance.

## GENOME ANNOUNCEMENT

Swine influenza virus (SIV), a member of the genus *Orthomyxovirus* (family *Orthomyxoviridae*), is a single-stranded, negative-sense RNA virus that causes an acute and highly contagious respiratory disease in swine. The pig can serve as a mixing vessel for the generation of genetically reassortant viruses because the porcine tracheal cells have receptors for both avian and human influenza viruses (1, 2). It has been reported that some new variants were generated by reassortment between avian and human viruses that occurred in pigs in nature (3, 4, 5), and the new variants have the potential to pose a serious threat to public health. Several sporadic human infections caused by swine-like viruses were reported (6, 7). A novel inﬂuenza A (H1N1) virus genome which contained human, swine, and avian virus genes emerged in the human population in 2009, and this pandemic strain may be derived from pigs (8, 9). Thus, it is important to enhance the surveillance of SIVs.

In this study, A/swine/Guangxi/BB1/2013 (H1N1) (GXBB1) was isolated from a sick pig in Guangxi, China. The eight genes of GXBB1 were amplified by reverse transcription (RT)-PCR using universal primers (10, 11). The amplified products were purified and cloned into the pMD-18T vector (TaKaRa) and sequenced (TaKaRa, Dalian, China) (12). Sequences were assembled and manually edited to generate the final full-length genome sequence.

The genome of the GXBB1 virus consisted of eight gene segments, which included polymerase (PB2, PB1, and PA), hemagglutinin (HA), nucleoprotein (NP), neuraminidase (NA), matrix protein (M), and nonstructural protein (NS) genes, respectively. The PB2 gene consisted of 2,280 nucleotides (nt), the PB1 gene 2,274 nt, the PA gene 2,150 nt, the HA gene 1,701 nt, the NP gene 1,515 nt, the NA gene 1,410 nt, the M gene 982 nt, and the NS gene 838 nt. The PSIQSR↓G in the HA cleavage site and 92 D in the NS1 site classified GXBB1 as a low pathogenic influenza virus. 190D, 220R, 225E, 226Q, and 228G (according to H3 numbering) in HA conferred preferential binding to the SAα-2,6-Gal receptor. The amino acid residues in NA associated with neuraminidase inhibitory drugs were conservative, which implied that GXBB1 might be sensitive to neuraminidase inhibitors, but S31N in M2 suggested that GXBB1 might be resistant to M2 ion channel inhibitors.

Phylogenetic analysis showed that the HA, NA, and M genes belonged to the European avian lineage. The PB2 and PA genes were both from a North American avian lineage. PB1 was a North American triple-reassortant SIV of human origin. The NP and NS genes were from a classical H1N1 swine lineage, and the PB2, PB1, PA, NS, and NP genes were derived from the reassortant H1N2 or H3N2 SIV. Phylogenetic analysis showed that the GXBB1 virus was a “human-swine-avian” triple-reassortant H1N1 virus, which was generated through gene reassortment between triple-reassortant H1N2 SIV or H3N2 SIV and European avian-like H1N1 SIV.

Our results demonstrated that the GXBB1 isolate is a triple-reassortant SIV whose genes were derived from avian, human, and swine origins.

### Nucleotide sequence accession numbers.

The genome sequence of A/swine/Guangxi/BB1/2013 (H1N1) was deposited in GenBank under the accession numbers KJ174942–KJ174949.
